# An Identification Method for Irregular Components Related to Terminal Blocks in Equipment Cabinet of Power Substation

**DOI:** 10.3390/s23187739

**Published:** 2023-09-07

**Authors:** Weiguo Cao, Zhong Chen, Xuhui Deng, Congying Wu, Tiecheng Li

**Affiliations:** 1 School of Electrical Engineering, Southeast University, Nanjing 210096, China; caoweiguo_seu@163.com; 2 Fuzhou Power Supply Branch, State Grid Fujian Power Company, Fuzhou 350001, China; 220202893@seu.edu.cn; 3 State Grid Economic and Technological Research Institute Co., Ltd., Biejing 100005, China; wucongying@chinasperi.sgcc.com.cn; 4 Power Science and Research Institute of State Grid Hebei Power Co., Beijing 430024, China; ltc8086@163.com

**Keywords:** small target detection, electrical cabinet, power substation, YOLOv7, differentiable binarization

## Abstract

Despite the continuous advancement of intelligent power substations, the terminal block components within equipment cabinet inspection work still often require loads of personnel. The repetitive documentary works not only lack efficiency but are also susceptible to inaccuracies introduced by substation personnel. To resolve the problem of lengthy, time-consuming inspections, a terminal block component detection and identification method is presented in this paper. The identification method is a multi-stage system that incorporates a streamlined version of You Only Look Once version 7 (YOLOv7), a fusion of YOLOv7 and differential binarization (DB), and the utilization of PaddleOCR. Firstly, the YOLOv7 Area-Oriented (YOLOv7-AO) model is developed to precisely locate the complete region of terminal blocks within substation scene images. The compact area extraction model rapidly cuts out the valid proportion of the input image. Furthermore, the DB segmentation head is integrated into the YOLOv7 model to effectively handle the densely arranged, irregularly shaped block components. To detect all the components within a target electrical cabinet of substation equipment, the YOLOv7 model with a differential binarization attention head (YOLOv7-DBAH) is proposed, integrating spatial and channel attention mechanisms. Finally, a general OCR algorithm is applied to the cropped-out instances after image distortion to match and record the component’s identity information. The experimental results show that the YOLOv7-AO model reaches high detection accuracy with good portability, gaining 4.45 times faster running speed. Moreover, the terminal block component detection results show that the YOLOv7-DBAH model achieves the highest evaluation metrics, increasing the F1-score from 0.83 to 0.89 and boosting the precision to over 0.91. The proposed method achieves the goal of terminal block component identification and can be applied in practical situations.

## 1. Introduction

Under the background of low-carbon development in energy industries [1], the operation and maintenance of power substations are progressing in a digital and intelligent direction [2,3]. Modern power grids, which involve smart grids, microgrids, and intelligent substations, require more digitized solutions or data-driven applications to improve operation flexibility and economic property [4,5]. Although the intelligent substation has realized the intelligence of the automation system, many tasks of construction or operation inspection for decades still require a lot of manual documenting work for unstructured forms of drawings, documents, and other data carriers in the manufacturing processes. It’s important to propose some solutions for repetitive and inefficient work.

The advancement of computer science and information technology has led to a significant improvement in artificial intelligence, particularly in the field of deep learning [6,7]. This progress has opened up new avenues for the automation of online monitoring and fault diagnosis at substations. Oliveira et al. [8] designed a remote monitoring system for substation bay construction with the goal of automated monitoring and management. Similarly, Zhao et al. [9] proposed a personal safety protective equipment detection model to remind employees who violated safety regulations during operations to take action, protecting them from electrical injuries. Yan et al. [10] compared six variants of the You Only Look Once (YOLO) [11,12,13] model, which served as object and violation detectors in the realistic substation construction site of different scenarios. Thanks to the algorithms’ open-source nature, the researchers face fewer obstacles to reproducing and applying the detection model and can focus on the adjustments and improvements to the concrete problems.

Huang et al. [14] identified the working state of isolation switches based on YOLO version 4 (YOLOv4) [15] through robot inspection images, even in cases of foggy or rainy weather. Similarly, Lu et al. [16] proposed a segmentation-based network to recognize the working state of the isolating switch in the traction substation; their experiment results suggest the pixel-level segmentation approach exhibits more fine-grained feature extraction capability. Huang et al. [17] also utilized image segmentation methods to separate the damper from its complex background to accurately recognize the damper rust status. Nassu et al. [18] designed a vision-based monitoring system for disconnect switches in distribution substations with multiple machine-learning techniques. In addition to scene images, infrared images also make contributions to equipment status recognition and fault diagnosis. Zheng et al. [19] proposed an improved Single Shot MultiBox Detector (SSD) [20] model to detect insulation status in substations, making full use of the thermographic diagnostics of insulators. To reduce the background interference in infrared images from substation scenes, the detection model is replaced by CenterNet with an Iresgroup structure to gain higher accuracy and better robustness in [21].

The inspection tasks associated with secondary system equipment, characterized by repetition and meticulousness and previously reliant on substantial manpower, can also benefit from technical support and automation. A recognition framework for knob gears in unattended substations is described in [22], in which key point extraction is an important part of calculating the correct angle under image noise and oblique view. Liu et al. [23] proposed a versatile pointer meter recognition system that exploits the Regions with Convolutional Neural Networks features (Faster R-CNN) [24] as a meter detector, image post-process methods as cropped image revision, and Hough transform as a pointer meter reader. The post-process includes feature correspondence algorithms and perspective transformation to increase the image quality. Deng et al. [25] selected the YOLO version 5 (YOLOv5) model for its faster running speed, and a segmentation network, DeepLabv3+ [26], was applied during the meter reading process to achieve better segment results on fine objects. Fan et al. [27] constructed their meter reader system on their self-built power meter dataset. Fan et al. further conducted their work on dual-pointer meters by means of improved segmentation models and a full meter recognition workflow, which largely expands the scope of its application. Wang et al. [28] integrated the mobile-friendly Vision Transformer (MobileViT) [29] module into the YOLOv7-tiny model to improve feature extraction and fusion ability for bounce lock status inspection tasks in substations. Despite the improvements to the backbone network part, the tested images of cabinet bounce locks all have plain backgrounds, and the target-bound locks are sparsely distributed in the cabinet.

Despite the notable advancements achieved through the integration of AI techniques in power substations, the utilization of AI in the digital modeling of secondary system data remains constrained. The current method of inspecting the physical wiring of protection screen cabinets in substations involves workers who manually inspect and compare the terminal block components to their original design. This process is hardly adequate for efficient construction and advanced applications for intelligent power substations, which are integral to modern power systems. That’s because digital modeling of physical devices, such as electrical wiring connection identification tasks, contains several sub-tasks, including element detection, text recognition, and connection recognition. Furthermore, tiny element target detection in complex scenarios presents significant challenges for the digital modeling of secondary systems in substations. Therefore, it’s of great value to devise a terminal block components detection and recognition system for digital modeling secondary systems in substations.

In this paper, we propose an innovative approach for the detection and identification of irregularly shaped physical components within terminal blocks located in power substation equipment cabinets. Our approach capitalizes on the YOLOv7 model as the initial network structure for both block area extraction and terminal block component detection. As the widely approved no-free-lunch theorems [30,31] pointed out, the performance of the YOLOv7 model is offset by our downstream tasks. Therefore, it’s necessary to improve the detection algorithm for better performance in terminal block component detection tasks. In the block area extraction task, we developed a streamlined version of YOLOv7 to swiftly locate complete terminal block regions while maintaining a higher processing speed. For the task of component detection, we retain the backbone network and feature fusion modules of YOLOv7, with primary enhancements focused on the prediction head via differentiable binarization segmentation. To achieve comprehensive component identity recognition, we employ the latest general OCR algorithm, the PaddleOCR tool [32], as the final step to match and consolidate identification results within our designed system.

The main contributions of this paper are as follows:

- To address the challenge of identifying minute, irregularly shaped component objects, we devise a three-stage system leveraging deep learning techniques. This system comprises terminal block area extraction, the detection of three categories of terminal block components, and element identity text recognition.
- In response to the distinctive regional characteristics of terminal blocks, we developed the YOLOv7 Area-Oriented (YOLOv7-AO) model for area extraction. This model omits the prediction branches and corresponding feature fusion modules that offer limited value. The streamlined regional detection model efficiently extracts block regions with reduced computational demands.
- To accommodate the densely arranged tags and cable markers within terminal blocks, we integrate differentiable binarization (DB) and an attention mechanism into the segmentation heads. This redesigned YOLOv7 model with a differentiable binarization attention head (YOLOv7-DBAH) significantly enhances element detection accuracy by producing results with precise boundaries.

The remainder of this paper is structured as follows. Section 2 provides a brief overview of the used terminal block components dataset. Section 3 explicitly describes the overall framework of block component detection and identification systems and introduces the model architecture of YOLOv7-AO and YOLOv7-DBH/DBAH models. Section 4 presents the corresponding model training process and the comparative experiments with other methods. Section 5 concludes this paper.

## 2. Materials

### 2.1. Datasets

#### 2.1.1. Dataset Acquisition

**Figure 1:**
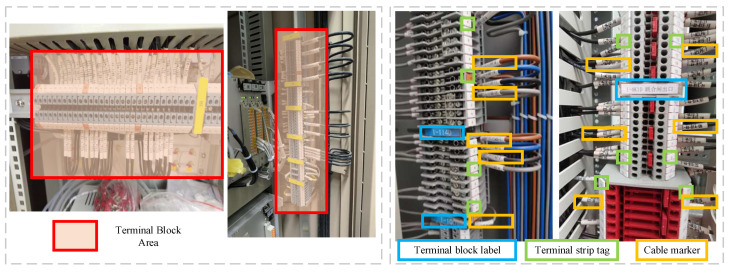
The overview of terminal block components appearances.

As of the present, no publicly accessible image dataset specifically catering to the task of identifying terminal block components is available. Hence, it becomes imperative for the subsequent detection models to establish a dataset characterized by both high quality and substantial volume. This image dataset is meticulously assembled through the utilization of high-resolution cameras, and its manual annotation is facilitated by employing the LabelImg tool. In order to ensure the clarity of each component instance, the filming distance from the target terminal block is subject to variations from the block’s actual size. Figure 1 exemplifies the appearance of key components featured in our paper, as depicted in image samples extracted from various power substations within our compiled dataset.

#### 2.1.2. Data Enhancement

Deep learning models necessitate a significant volume of data to effectively harness the automatic feature extraction capabilities. Beyond the sheer scale of the dataset, the assortment and proportion of distinct categories also exert influence on the ultimate outcomes of model training in the context of a multi-class detection problem. The statistical findings pertaining to the dataset are exhaustively detailed in Table 1. Instances that exhibit subpar quality or an oblique perspective are judiciously eliminated during the dataset generation phase to preempt the propagation of misleading labels. Evidently, the tally of labels assigned to terminal blocks is comparatively lower than the counts for the remaining two components due to the specific manner in which sub-area markings are practically utilized.

To cope with the challenge from the data source, a series of data enhancement operations are applied. Along with the subdivision of the training, validation, and testing datasets, numerous basic online enhancement operations (color distortion, image flipping and rotation, and noise addition) [10] are used during the training stage. Moreover, a mosaic data enhancement strategy involving image slicing, cropping, and combination [27] is utilized for the block area location model, which allows the area detection model to function well in datasets of a smaller scale.

## 3. Methods

**Figure 2:**
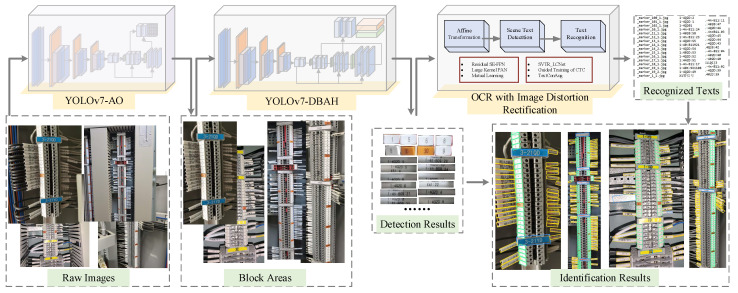
The overall framework of terminal block components identification method. Blue box indicates Terminal block label; green box indicates Terminal strip tag; yellow box indicates Cable marker; red box indicates Terminal Block Area.

The comprehensive process of the terminal block component identification method is visually depicted in Figure 2. The identification method consists of the following steps. The initial step involves the location and extraction of the terminal block region in images by the YOLOv7-AO model to filter out unrelated backgrounds. Subsequently, three kinds of terminal components, namely terminal block labels, terminal strip tags, and cable markers, are detected within the terminal block area based on the YOLOv7-DBAH model and then cropped out for text recognition. A conventional box formation method is applied for segmentation output. To refine the detection results for each individual instance, an image distortion rectification technique founded on affine transformation is employed. Moreover, the label text associated with each element is recognized via OCR algorithms, thereby achieving the terminal component identification task. The following section introduces the details of the proposed model’s structure and procedure.

### 3.1. Terminal Block Area Extraction

**Figure 3:**
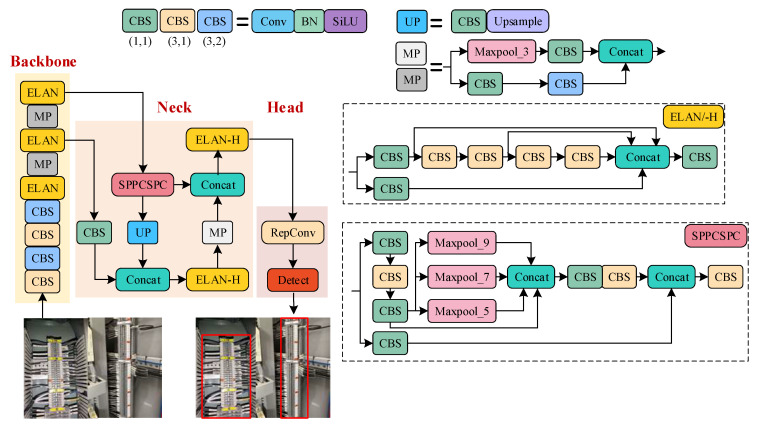
The structure of YOLOv7-AO model. The red boxes represent the targets detected by the YOLOv7-AO model.

In the physical layout of terminal blocks within equipment cabinets on-site, a significant proportion of images predominantly capture the entirety of a single terminal block. Consequently, the number of regions of interest (ROI) is relatively limited. The detection of the terminal block area location is a typical large-scale object detection problem that removes the image portion of unrelated devices and cabinet background for subsequent tasks in the meantime. Therefore, building upon the conventional YOLOv7 model, this section develops a YOLOv7-AO model that is specifically designed to detect large-scale terminal block areas. The model’s structure is elaborated in Figure 3. The red boxes represent the targets detected by the YOLOv7-AO model.

By leveraging a backbone network, the YOLOv7-AO model is able to extract features of different scales from input images, with a main focus on high-level features for the purpose of locating terminal blocks. The streamlined YOLOv7-AO model is capable of quickly locating large targets and filtering irrelevant background noise, thereby enhancing the overall efficiency of the identification process.

The training loss $L_{AO}$ of YOLOv7-AO comprises of boundary regression loss $L_{box}$, classification loss $L_{cls}$, and object loss $L_{obj}$, as shown in Equation (1).

(1) $$L_{AO}=L_{box}+L_{cls}+L_{obj}$$

Both the classification loss $L_{cls}$ and object loss $L_{obj}$ adopt the binary cross-entropy (BCE) loss function, as shown in Equation (2).

(2) $$L_{BCE}\left(y,f\left(x\right)\right)=-y\ln f\left(x\right)-\left(1-y\right)\ln\left(1-f\left(x\right)\right)$$

where $f\left(x\right)$ indicates the predicted outputs, y indicate the ground-truth labels.

The boundary regression loss $L_{box}$ is calculated using complete intersection over union (CIoU) loss in a much more complex way to obtain accurate target boundaries, which is expressed in Equation (3).

(3) $$\left\{\begin{array}{l} L_{CIoU}=1-I_{IoU}+\frac{d_{o}^{2}}{d_{c}^{2}}+\frac{\nu^{2}}{1-I_{IoU}+\nu }\\ I_{IoU}=\frac{\left|b\cap b^{gt}\right|}{\left|b\cup b^{gt}\right|}\\ \nu =\frac{4}{\pi^{2}}{\left(\arctan\frac{w_{gt}}{h_{gt}}-\arctan\frac{w}{h}\right)}^{2} \end{array}\right.$$

where $b,\;b^{gt}$ indicate the predicted boundary box and its ground-truth label respectively, $w,\;h,\;w^{gt},\;h^{gt}$ indicate the width, height of predicted boundary box and its ground-truth label respectively, $d_{o}^{2}$ indicates the distance between the centers of predicted and labelled box, $d_{c}^{2}$ indicates the largest distance between the boundaries of predicted and labelled box, ν indicates the penalty of aspect ratio gap.

### 3.2. Terminal Block Components Detection

**Figure 4:**
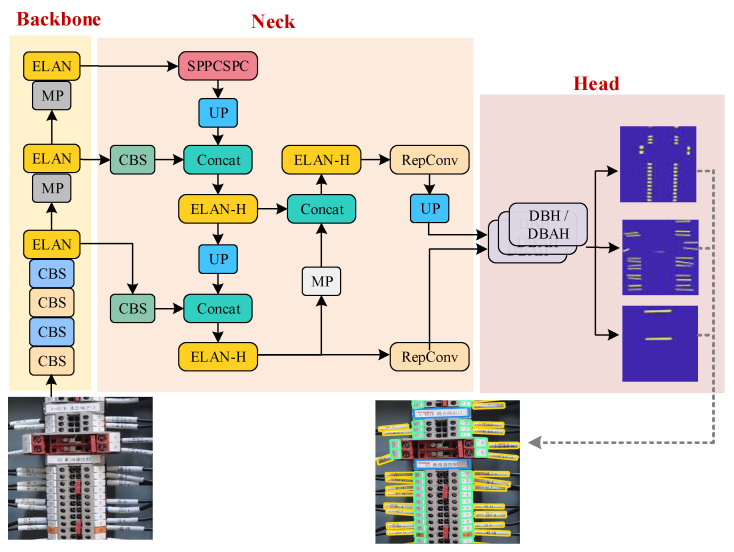
The structure of YOLOv7-DBH/DBAH model. Blue box indicates Terminal block label; green box indicates Terminal strip tag; yellow box indicates Cable marker; red box indicates Terminal Block Area.

This section presents the Yolov7-DBAH model as a novel approach for detecting terminal block components. The proposed model is an adaptation of the YOLOv7 model, which is specifically designed for detecting small-sized targets. The original detection head of the YOLOv7 model is replaced with a segmentation head that supports differentiable binarization [33] for detecting multiple target components with irregular shapes simultaneously. To further enhance the performance of the segmentation head, channel attention, and spatial attention modules are integrated, leading to the DBAH segmentation head. The YOLOv7-DBAH model is constructed to detect all three categories of terminal block components, and the overall network structure is shown in Figure 4.

The backbone and feature fusion module of the Yolov7-DBAH model are consistent with the conventional YOLOv7 model. The multi-channel features of different targets can be extracted from the terminal block region. In view of the small-scale and densely packed nature of terminal strip tags and cable markers, the original high-level prediction branch is deemed insufficient and is thus removed, leaving only the mid-level and low-level prediction branches. Unlike the YOLOv7 model, where the two prediction branches operate independently to produce detection results, the DBH/DBAH segmentation heads receive mid-level and low-level feature maps after feature map size alignment.

The YOLOv7-DBAH model also employs BCE loss as its training loss, which is a commonly used loss function in classification tasks. The terminal block components, namely terminal block labels, terminal strip tags, and cable markers, correspond to three groups of approximate binarization maps, probability maps, and threshold maps. The BCE loss is applied to the approximate binarization map loss and probability map loss, while the L1 loss function is used for the threshold map. Each group of outcome maps is supervised and contributes to the calculation of its respective component training loss. These component losses are then aggregated for gradient descent backpropagation.

(4) $$L_{p}=\sum_{m}^{M}L_{BCE}\left(y_{m}^{p},f_{m}^{p}\left(x\right)\right)$$

(5) $$L_{b}=\sum_{m}^{M}L_{BCE}\left(y_{m}^{b},f_{m}^{b}\left(x\right)\right)$$

(6) $$L_{t}=\sum_{m}^{M}\left|y_{m}^{t}-f_{m}^{t}\left(x\right)\right|$$

(7) $$L_{agg}=L_{p}+\lambda_{b}L_{b}+\lambda_{t}L_{t}$$

where $f_{m}^{p}\left(x\right),f_{m}^{b}\left(x\right),f_{m}^{t}\left(x\right)$ indicate the predicted outputs of probability maps, approximate binarization maps, and threshold maps for each target category, respectively; $y_{m}^{p},y_{m}^{b},y_{m}^{t}$ indicate the ground-truth labels of the probability maps, approximate binarization maps, and threshold maps for each target category, respectively; $\lambda_{b}$ denotes scale factor of the binarization maps, set to 1; $\lambda_{t}$ denotes scale factor of the threshold maps, set to 10.

The differentiable binarization technique involves the initial prediction of probability and threshold maps, which are subsequently utilized to produce a binarization map output. The model used in this process is equipped with three DBH/DBAH heads, which correspond to the three terminal block component categories that require identification.

**Figure 5:**
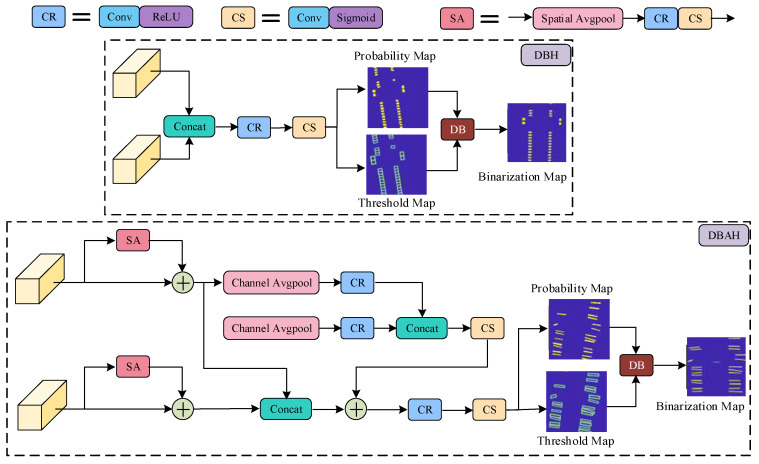
The structure of DBH/DBAH segmentation head.

The DB segmentation head converts the boundary box regression problem into a segmentation problem, resulting in a more precise contour of the actual boundary of terminal block components. This approach effectively resolves the problem of image distortion in target object detection. To further improve the detection of multiple classes simultaneously, a DBAH segmentation head is developed by integrating spatial and channel attention mechanisms into the basic DBH segmentation head structure. The structure of both DBH and DBAH segmentation heads is illustrated in Figure 5.

The DBH segmentation head is characterized by a straightforward structure. It involves the direct concatenation of two sets of feature maps, which are subsequently fused with a 3 × 3 convolutional layer with ReLU activation and a 1 × 1 convolutional layer with Sigmoid activation. The probability map and threshold map are predicted from fused features and are then utilized to produce a binarization map via a preset differentiable binarization function. From the binarization results, the boundaries of each instance are easy to obtain and approximated by a rotating rectangular box, bringing a unified and organized format of detection results.

By adding Spatial Attention (SA) and Channel Attention (CA) modules to the DBH segmentation head, the DBAH segmentation head strengthens the relevant features for each target category. The DBAH segmentation head utilizes independent spatial attention mechanisms to predict the spatial attention weights related to the target class before concatenating sets of feature maps. The CA module concatenates the attention vectors of two sets of feature maps and further calculates the global channel attention vector. The feature maps are then concatenated and fused with the channel attention vectors. The enhanced feature maps are subject to two convolutional layers: the generating target probability map, the threshold map, and the binarization map. These outcome maps are then utilized to obtain detection results in the same way as DBH heads.

The standard binarization process is expressed in Equation (8). While this standard binarization operation is intuitive and fast, its non-differentiable nature renders it unsuitable for joint optimization within the neural network.

(8) $$f_{sb}\left(p,T_{b}\right)=\left\{\begin{array}{l} 1,\;p\geq T_{b}\\ 0,\;p<T_{b} \end{array}\right.$$

where p is the coordinate point of the probability map, $T_{b}$ is the binarization threshold.

The differentiable binarization process is expressed in Equation (9). Compared with the standard binarization operation, the introduction of the exponential function imparts differentiability to the binarization operation. Furthermore, scale factors are integrated to make the function curve approach the step function, i.e., the standard binarization function, so as to reach the goal of the binarization operation.

(9) $$f_{db}\left(p,t\right)=\frac{1}{1+e^{-k_{b}\left(p-t\right)}}$$

where t is the coordinate point of the threshold map, $k_{b}$ indicates the scale factor for differentiable binarization, empirically set to 50.

The differential of a differentiable binarization operation can be calculated through the BCE loss function for binarization maps. With the positive labels in the foreground and the negative labels in the background, where the loss $L^{+}$ is for positive labels and $L^{-}$ for negative labels, the corresponding differential of the losses for probability maps can be expressed in Equation (10). The differential for threshold maps can be obtained in a similar manner.

(10) $$\left\{\begin{array}{l} \frac{\delta L^{+}}{\delta p}=-k_{b}f_{db}\left(p,t\right)e^{-k_{b}\left(p-t\right)}\\ \frac{\delta L^{-}}{\delta p}=k_{b}f_{db}\left(p,t\right) \end{array}\right.$$

**Figure 6:**
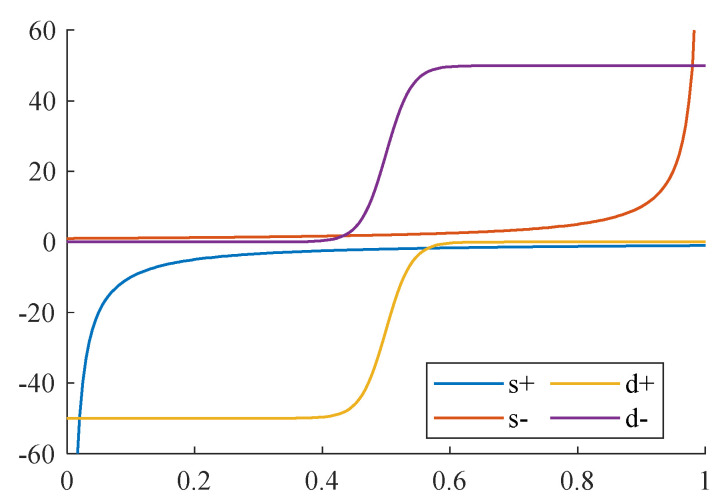
The differential of losses of standard and differentiable binarization. The ‘s+’ and ‘s−’ are respectively the differential of positive and negative labels of standard binarization; The ‘d+’ and ‘d−’ are respectively the differential of positive and negative labels of differentiable binarization.

Accordingly, the differentials of the BCE losses of standard binarization and differentiable binarization are also shown in Figure 6. From the comparison, it can be perceived that the differential of training losses for boundary points or ambiguous points is amplified, guiding the network to correct the wrong outputs during the gradient descent backpropagation. In most cases where points are misclassified, their derivatives are greater, which can give effective feedback for backpropagation. Moreover, both the least upper bound and the greatest lower bound of the differential are constrained by the scale factor and binarization output, reducing the impact of extremely large or small values.

To alleviate the computational load associated with exponential operations during the inference stage, a new binarization function is designed to be an approximate substitute for the original differentiable binarization function (set to 50), as shown in Equation (11).

(11) $$f_{adb}\left(p,t\right)=\left\{\begin{array}{l} 0,\;\;\;\;\;p-t<-0.075\\ \frac{p-t}{0.15}+0.5,\;\left|p-t\right|\leq 0.075\\ 1,\;\;\;\;\;p-t>0.075 \end{array}\right.$$

**Figure 7:**
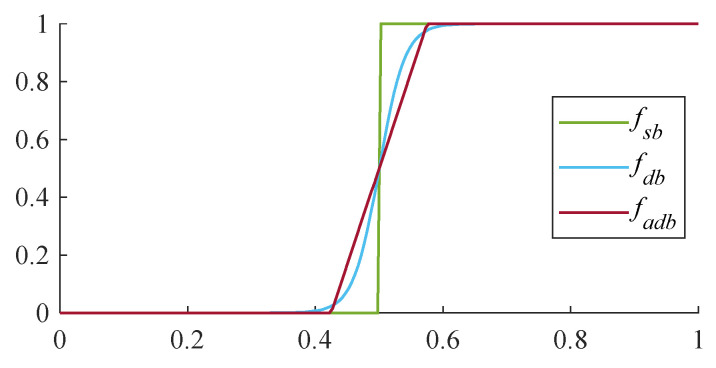
The comparison of binarization methods.

The comparison of the three above-mentioned binarization functions is presented in Figure 7. As shown in the figure, the differentiable binarization function can effectively mimic the standard binarization function. Even the approximate differentiable binarization function designed to accelerate computation can also accomplish the segmentation task of probability maps and obtain discriminating boundaries of instances with lower computational complexity.

### 3.3. Text Recognition with Image Distortion Correction

In this section, we make use of the PaddleOCR tool, which is comprised of text detection, direction rectification, and text recognition modules. The OCR system is characterized by its lightweight network structure while still providing a complete set of recognition functions. It is noteworthy that the PaddleOCR system can accurately recognize input text even when it is misaligned or relatively small compared with the component area. To address the issue of perspective distortion, a correction operation based on affine transformation is implemented individually for each instance before being fed into the OCR system. Considering the adoption of a standardized and uniform text font, the PaddleOCR system with distortion correction is deemed sufficient for the scene-text recognition task.

The marking texts on terminal block components encompass the device type, ID tag, and other relevant detailed information, which serve to convey electrical wiring contexts in an intuitive manner. The text recognition for detected components constitutes the conclusive stage in the identification method for terminal block components and is of vital importance for the documentation of on-site devices and wiring connections.

## 4. Results and Discussion

### 4.1. Experimental Setup

#### 4.1.1. Experiment Platform

The experimental environment is detailed as follows: Windows 10, Python 3.8, Pytorch 1.9, and PaddleOCR 2.6. The computing workstation for model training is configured with a CPU (AMD Ryzen 9 5900X 12-Core), a GPU (NVIDIA GeForce RTX 3080), and RAM (32 GB). This training platform is sufficient for model training under the following training settings. Computational environments with similar or higher GPU configurations should be suitable for the proposed methods. It’s not recommended to train the networks in environments with a GPU of less than 4 GB. The computing device for inference speed tests is the NVIDIA Xavier NX. The computation requirements for testing the trained models are open to devices with a greatly wider selection range.

#### 4.1.2. Experiment Settings

For the task of terminal block area extraction, stochastic gradient descent (SGD) with a momentum of 0.9 is employed for network optimization. The learning rate is initially set to 1 × 10^−2^ with a weight decay of 5 × 10^−4^. The total epoch is set to 270 with a batch size of 16.

For the task of terminal block component detection, the optimizer is still SGD with a momentum of 0.9. The initial learning rate is decreased to 1 × 10^−3^ with the same weight decay of 5 × 10^−4^. The training total epoch is increased to 500 with a larger batch size of 32.

#### 4.1.3. Evaluation Metrics

Various evaluation metrics are employed in this paper to comprehensively assess the detection performance of different models. For the detection tasks, precision, recall, and the F1-score are the classic metrics, which can be deduced from four basic statistical indicators, true positive (TP), true negative (TN), false positive (FP), and false negative (FN). Precision measures the proportion of correct positive samples in detected samples, while recall measures the ratio of correct samples in actual positive samples. As the harmonic average of precision and recall, the F1-score reflects precision and recall in a comprehensive way.

(12) $$\mathrm{Precison}\;=\;\frac{TP}{TP+FP}$$

(13) $$\mathrm{Recall}\;=\;\frac{TP}{TP+FN}$$

(14) $$F1\;=\;\frac{2\times \mathrm{Recall}\times \mathrm{Precison}}{\mathrm{Recall}+\mathrm{Precison}}$$

In addition to the three indicators, mean average precision (mAP) and frames per second (FPS) are commonly used in objection detection problems. The mAP measures the average performance in all target categories at different confidence levels. Furthermore, the FPS indicator measures the running speed of a deep learning model, reflecting the complexity of a network.

(15) $$\mathrm{mAP}\;=\;\frac{1}{Q}\sum_{q=1}^{Q}\int_{0}^{1}P_{q}\left(R\right)dR$$

where Q is category number, set to 3 (the terminal block labels, cable markers, and terminal strip tags) in this paper; $P_{q}\left(R\right)$ is the Precision Recall Curve (PRC) of a category.

### 4.2. Experimental Results

#### 4.2.1. Terminal Block Area Exaction Results

**Figure 8:**
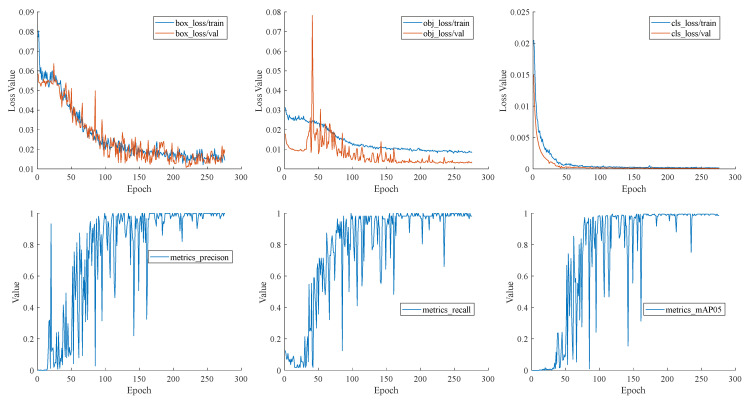
Training process of YOLOv7-AO.

Figure 8 illustrates the training process of the YOLOv7-AO model, highlighting the differing convergence behaviors of different loss components. The classification loss (cls_loss) exhibits the fastest convergence speed and the smoothest loss curve, owing to the limited number of target categories in the terminal block areas. The object loss (obj_loss) experiences the most fluctuations in the early stage of training but gradually converges after 180 rounds in the later stage. In contrast, the boundary regression loss (box_loss) curve fluctuates more stably.

The convergence of the training loss is observed to occur towards the later stages of training, indicating that the model has attained the desired level of convergence within the expected time. This observation is further supported by the mAP and other metric curves, which exhibit a similar pattern. All the evaluation metrics approach the expected level after 160 rounds. Despite some oscillations around 200–250 rounds, they are eventually resolved, and the model converges.

**Figure 9:**
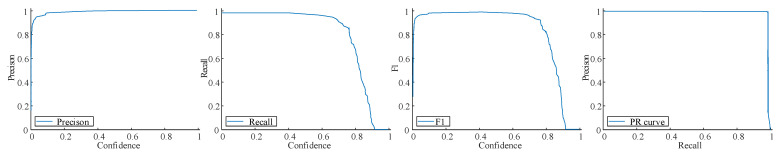
The metrics of Precision, Recall, F1-score, and PR-curve.

Following training, the YOLOv7-AO model is evaluated for its precision, recall, F1-score, and PR curves after training, as presented in Figure 9. The evaluation results indicate that all target curves meet the expected standards. The precision curve surpasses 0.8 before the confidence level of 0.1, while the recall curve exhibits a sharp decline only after the confidence level of 0.75. Moreover, the PR curve is positioned close to the upper right corner of the coordinate axis, which confirms the effectiveness of the YOLOv7-AO model in accurately locating terminal block areas.

To validate the superiority of the YOLOv7-AO model, a comparative experiment is carried out to assess the effectiveness and inference speed of the standard YOLOv7 model and the YOLOv7 tiny model using identical test data. The comparative experimental results are presented in Table 2. The experimental results indicate that the YOLOv7-AO model performs better than the standard YOLOv7 model in terms of inference speed, suggesting that the proposed model has a faster deployment speed. Moreover, the YOLOv7-AO model exhibits a regional detection precision that is comparable to that of the standard YOLOv7 model and superior to the simplified version, the YOLOv7-tiny model.

#### 4.2.2. Terminal Block Components Detection Results

**Figure 10:**
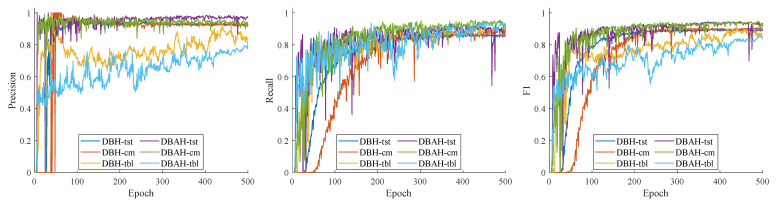
Training process of YOLOv7-DBH and YOLOv7-DBAH model.

The training procedure of the YOLOv7-DBH/DBAH model is evaluated based on precision, recall, and F1-score, as shown in Figure 10. The target components analyzed are terminal strip tags (tst), cable markers (cm), and terminal block labels (tbl). The results indicate that the terminal block label exhibited a significantly greater fluctuation amplitude compared with the other two components, which are detected by both DBH and DBAH segmentation heads.

The fluctuations observed during training can be attributed to the relatively lower number of terminal block labels compared with the other components. In addition, it is noteworthy that terminal block labels exhibit greater length and width dimensions than terminal strip tags and cable markers. Unlike the densely packed other components, there are few or no instances of closely arranged terminal block labels, which may account for the slightly lower precision and F1-score. Nevertheless, the performance of the model remains adequate for practical applications.

The comparison of the training procedures for the DBAH and DBH segmentation heads reveals that the evaluation metrics for terminal strip tags and cable markers are comparable. The DBAH model exhibits marginally superior recall and precision for cable markers, while the DBH model displays nearly identical values for both metrics. The inclusion of supplementary attention mechanisms in the DBAH segmentation head leads to greater fluctuations in the evaluation metric during the initial stages of training. However, as the training procedure progressed towards convergence, the fluctuations gradually diminished and converged to a higher level.

The DBH segmentation head benefits from its simpler model structure and exhibits stable performance during the training stage, with only minor fluctuations observed. Specifically, the precision curve experiences a sudden drop before 50 rounds, while the recall curve experiences a sudden drop around 300 rounds. However, the metric curve oscillates and quickly recovers to its original level. In terms of the evaluation metric performance after convergence, the precision of the DBAH segmentation head is comparable to that of the DBH segmentation head. On the other hand, the DBAH segmentation head outperforms the DBH segmentation head in terms of recall, particularly for terminal strip tags and cable markers. This advantage is also reflected in the F1-score performance.

**Figure 11:**
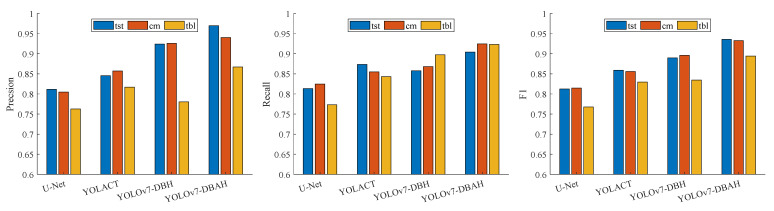
The Comparison of segmentation networks.

To assess the segmentation performance of the YOLOv7 DBAH model, comparative experiments with different segmentation networks are carried out using the same test data. The experimental results are tabulated in Table 3. The proposed YOLOv7-DBAH obtains better accuracy, but it can appear to fall short in terms of time cost, which is due to the fact that the new module incurs more cost. Meanwhile, the comparative experiment results for each subcategory are illustrated in Figure 11.

The above results of the experiment reveal that the YOLOv7-DBAH model exhibits the most superior performance in terms of the average evaluation outcomes for the three types of components. Even the lowest metric value exceeded 0.91. The YOLOv7-DBH model and YOLACT model are ranked in the second tier, with evaluation metrics hovering around 0.85. The classic segmentation model, U-Net, exhibits evaluation results of approximately 0.80.

The YOLOv7-DBH model outperforms benchmark segmentation models in terms of terminal blocks. Meanwhile, the YOLOv7-DBAH model, with its integrated spatial and channel attention mechanisms, enhances feature extraction for target-related components, leading to optimal segmentation performance. This mechanism aids in learning features relevant to target components, ultimately resulting in improved segmentation performance. The DBAH segmentation head, which is constructed based on attention mechanisms, has the potential to enhance the feature extraction ability in the context of unbalanced positive and negative samples. While the YOLOv7-DBH model fails to attain a higher F1-score in the terminal block label target compared with the YOLACT and U-Net models. Despite this, the YOLOv7-DBH model outperforms the YOLACT model in the average evaluation metrics, mainly due to its superior performance in terminal strip tags and cable markers.

#### 4.2.3. Visualization Analysis of Terminal Block Components Identification

**Figure 12:**
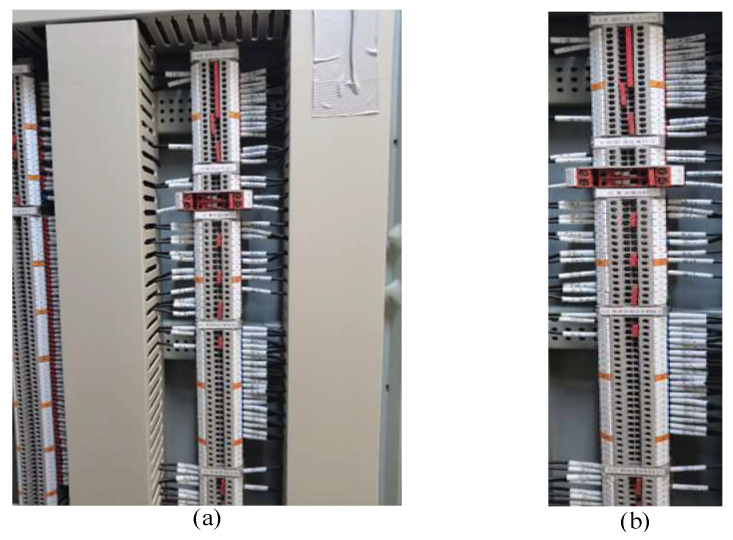
The input image and area location results. (**a**) is the raw image, (**b**) is extracted terminal block area for subsequent detection task.

**Figure 13:**
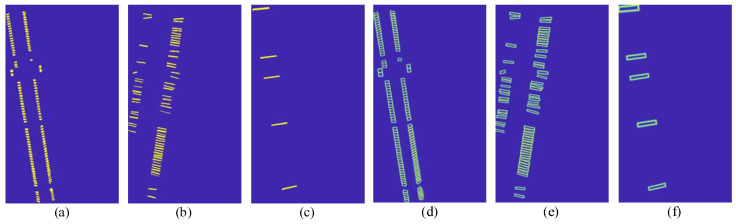
The outcome probability maps and threshold maps. (**a**–**c**) are the probability maps; (**d**–**f**) are the threshold maps; (**a**,**d**) belong to terminal strip tags, (**b**,**e**) belong to cable markers, (**c**,**f**) belong to terminal block labels.

**Figure 14:**
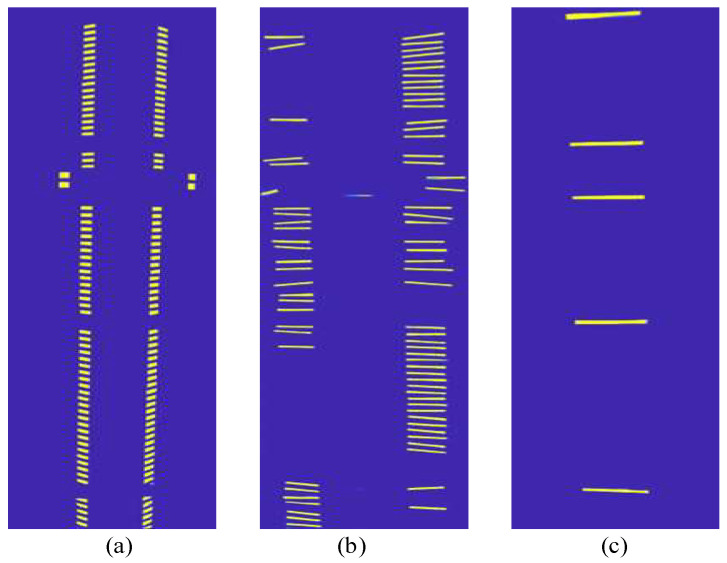
The outcome binarization maps. (**a**) belongs to terminal strip tags, (**b**) belongs to cable markers, (**c**) belongs to terminal block labels.

**Figure 15:**
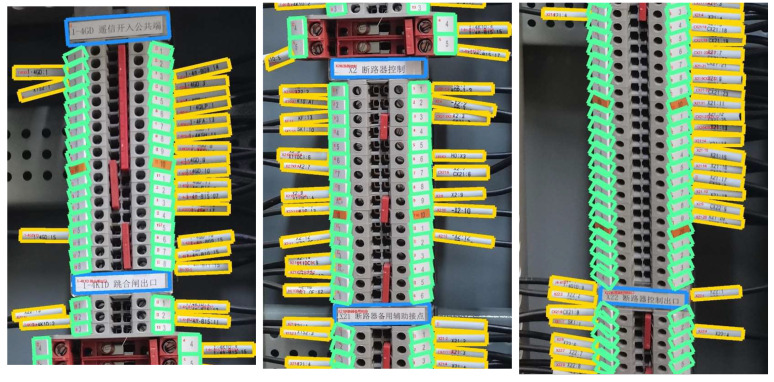
Display of the identification results. Blue box indicates Terminal block label; green box indicates Terminal strip tag; yellow box indicates Cable marker; red box indicates Terminal Block Area.

In this section, the essential steps in the identification methodology are presented through a typical terminal block image. The main process and key intermediate feature maps of terminal block components are illustrated in Figure 12, Figure 13, Figure 14 and Figure 15.

The outcome of the positioning task performed by the YOLOv7-AO model on the target terminal block region is presented in Figure 12. The model successfully excludes the irrelevant background region and accurately identifies the terminal block of interest. The model effectively handles the occlusion and distortion issues present in the terminal block located on the left side of the image.

Figure 13 and Figure 14 present probability maps, threshold maps, and binarization maps of terminal strip tags, cable markers, and terminal block labels processed by the DBAH head. The transformation of probability and threshold maps involves scaling and alignment operations that may introduce a slight asymmetry in the image. The results of the differentiable binarization process are adjusted to align with the input image.

By comparing the probability maps and binarization maps, it can be inferred that gaps between adjacent instances are of vital importance to identifying the densely arranged terminal strip tags or cable markers. The adaptive threshold processing method utilized in probability maps facilitates clear identification of such gaps. Besides, the threshold map prediction for terminal block labels can be omitted in the inference stage due to the limited number and wide spacing of such labels. The probability maps can be directly used to replace binarization maps without significant loss in precision.

In Figure 15, the detection bounding boxes for target objects are depicted, with the identification information of each instance annotated in red text. The detection task of terminal block labels, terminal strip tags, and cable markers exhibits satisfactory precision and recall performance, with boundary boxes closely resembling the actual target shape. It gives advantages to the item text recognition task. In spite of this, due to the curved shape and filming perspective of cable markers, the final text recognition results still require correction with electrical background knowledge during the implementation stage to obtain more precise text information.

## 5. Conclusions

In this paper, a multi-step identification method utilizing various deep learning techniques is proposed to address the detection challenges posed by small and densely arranged terminal block components. The method involves the use of a modified YOLOv7-AO area extraction model and an enhanced YOLOv7-DBAH detection model in the first and second stages of the system, respectively. The YOLOv7-AO model obtains a comparable accuracy performance with the standard YOLOv7 algorithm yet 4.45 times faster inference speed, dramatically decreasing the computation time of terminal block location. The block region extraction results suggest that the YOLOv7-AO model is well-suited for real-time applications, such as terminal block component area positioning tasks for video streams. The YOLOv7-DBAH model has demonstrated significant improvements in all evaluation metrics of the three terminal block components. This includes an increase in precision and recall, which are already higher than those of the YOLACT model, to over 0.91. Moreover, the F1-score of the terminal block label, which was initially only 0.83, has been increased to more than 0.89.

Future work would focus on improvements to the terminal block component identification accuracy and speed. The scene video of the terminal blocks can provide more complete information about their components. Analyzing the content of adjacent video frames can potentially enhance the accuracy of target identification and their wiring connections. This advancement could significantly improve the efficiency of substation inspection work by providing a more complete and contextual understanding of the terminal block components.

## Figures and Tables

**Table 1:** The statistics of terminal block components dataset.

	Train	Valid	Test
Images	1306	194	194
Terminal block label	2661	346	352
Terminal strip tag	25,836	1621	1635
Cable marker	19,894	1386	1403

**Table 2:** The comparison of area location models.

	mAP@0.5:0.95	FPS
YOLOv7	0.816	16.7
YOLOv7-tiny	0.746	68.5
YOLOv7-AO	0.809	74.3

**Table 3:** The comparison of average results of segmentation models.

	Precision	Recall	F1	Time Cost (h)
U-Net	0.7930	0.8037	0.7983	1.21
YOLACT	0.8398	0.8571	0.8483	4.52
YOLOv7-DBH	0.8768	0.8743	0.8735	4.61
YOLOv7-DBAH	0.9257	0.9173	0.9208	5.27

## Data Availability

Not applicable.
